# Incidence and Predictors of Catheterization-Related Cerebral Infarction on Diffusion-Weighted Magnetic Resonance Imaging

**DOI:** 10.1155/2016/6052125

**Published:** 2016-04-05

**Authors:** Yusuke Morita, Takao Kato, Mitsumasa Okano, Kanae Suu, Masahiro Kimura, Eri Minamino-Muta, Eisaku Nakane, Toshiaki Izumi, Shoichi Miyamoto, Tetsuya Haruna, Koji Ueyama, Moriaki Inoko

**Affiliations:** Cardiovascular Center, Tazuke Kofukai Medical Research Institute, Kitano Hospital, Osaka 530-8480, Japan

## Abstract

*Introduction*. The aim of this study was to examine the incidence and risk factors of catheterization-related CI in the contemporary era, using diffusion-weighted magnetic resonance imaging.* Methods*. We retrospectively analyzed consecutive 84 patients who underwent MRI (magnetic resonance imaging) after 2.81 ± 2.4 days (mean ± SD) of catheterization via aortic arch. We categorized the patients by the presence or absence of acute CI determined by diffusion-weighted MRI and analyzed the incidence and predictors.* Results*. Of 84 patients that underwent MRI after catheterization, acute CI was determined in 27 (32.1%) patients. In univariate analysis, dyslipidemia, age, coronary artery disease, antiplatelet agents, number of catheters used, urgent settings, and interventional procedures were significantly different. Multivariate analysis revealed dyslipidemia (odds ratio [OR], 4.46; 95% confidence interval [CI], 1.41–16.03; *p* = 0.01), higher age (OR, 1.09; 95% CI, 1.007–1.19; *p* = 0.03), and the number of catheters used (OR, 2.21; 95% CI, 1.21–4.36; *p* = 0.01) as independent predictors of the incidence of catheterization-related acute CI.* Conclusions*. Dyslipidemia, higher age, and number of catheters used were independent predictors for acute CI after catheterization. These findings imply that managing dyslipidemia and comprehensive planning to minimize the numbers of catheters are important.

## 1. Introduction

Catheterization is an established procedure for evaluation and intervention in cardiac and peripheral artery diseases. Catheterization-related acute stroke is a rare (reported incidence, 0.44%) but potentially devastating complication associated with high comorbidity and mortality [1–3]. Acute stroke associated with catheterization is classified into two major categories: symptomatic cerebral infarction (CI) and hemorrhagic stroke; respective rates are reported as 0.22% and 0.21% in retrospective study, and the causative mechanisms are potentially different [1]. Catheterization-related CI is caused mainly by dislodgement of atherosclerotic debris from the aorta during catheterization [4]. Furthermore, air embolism [5], thrombus formation in or on the surface of the catheter, changes of devices in the catheters, and hemodynamic compromise [6] are capable of causing acute cerebral injury during catheterization.

On the other hand, unperceived acute CI after catheterization could be detected at an unexpectedly high rate on diffusion-weighted (DW) magnetic resonance imaging (MRI), as described in 4.9–34.7% of patients [7–15]. Combination of DW imaging and apparent coefficient (ADC) maps on MRI is commonly accepted technique for assessment of acute CI [16, 17]. CI is reported to be a cause of decline in cognitive function, even if there were no symptoms [18]. While temporal trends in catheterization have changed, such as increased adoption of the radial approach and complex percutaneous coronary intervention [2, 19, 20], recently, patients undergoing catheterization have been older and at greater risk of atherosclerosis [20]. Here, we present an analysis of risk factors and current incidence for catheterization-related CI detected using DW MRI, as well as the features of MRI findings. In this retrospective study, we investigated patients considered at high risk, including those suspected of acute stroke, those who underwent catheterization prior to cardiovascular surgery, and those who underwent urgent catheterization.

## 2. Methods

### 2.1. Study Population

A total of 1237 patients underwent catheterization via the aortic arch at the Cardiovascular Center, Kitano Hospital, between June 2010 and June 2012. Of these, we retrospectively identified 84 patients who underwent MRI after the catheterization at all, excluding those who underwent MRI more than 14 days after the procedure. Because the decrease in apparent diffusion coefficient (ADC) signals caused by acute CI was presumed to have returned to the baseline after several weeks [21], we used retrieval software and retrospectively reviewed medical records for each patient. The mean time period after the catheterization at which MRI was performed was 2.81 ± 2.4 days (mean ± SD). The reasons for MRI were shown in Table 1. Among all the patients, 26 patients (31%) were candidates for coronary artery bypass grafting, 15 patients (15.8%) for valvular surgery, 10 patients (11.9%) for assessments of symptoms, and 8 patients (9.6%) for peripheral artery or aortic surgery. Diagnostic and interventional procedures were performed according to current guidelines [22–25]. The research protocol was approved by the Institutional Review Board of Kitano Hospital according to the ethical guidelines of the 1975 Declaration of Helsinki. Since this is a retrospective study, the consent was not obtained and patient records/information was anonymized and deidentified prior to analysis.

### 2.2. Study Protocol

A medical history was documented in each patient prior to the catheterization procedure, which was performed by cardiologists. The following information was retrieved for each patient: DW MRI findings, catheterization procedure, left ventricular ejection fraction (LVEF) on echocardiography, and preprocedural medication. Baseline clinical characteristics include diabetes, smoking, dyslipidemia, family history of coronary artery disease (CAD), presence of CAD, prior stroke, myocardial infarction, peripheral vascular disease (PVD), renal impairment, and persistent atrial fibrillation (AF). Hypertension was defined as systolic blood pressure ≧140 mmHg, diastolic blood pressure ≧90 mmHg, or the use of antihypertensive drugs. Dyslipidemia was defined as either low-density lipoprotein cholesterol (LDL) ≧140 mg/dL or the use of statin. Renal impairment was defined as creatinine ≧1.5 mg/dL. CAD was defined as a diameter stenosis of 70% or more in at least one major epicardial artery by coronary angiography at the time of the study or earlier. CAD included myocardial infarction. Severe aortic stenosis was defined as either an aortic valve area ≦1.0 cm^2^, mean pressure gradient ≧40 mmHg, or aortic jet velocity ≧4.0 m/s according to the guideline [23].

### 2.3. Catheterization Procedures

Catheterization was undertaken by senior cardiologists in the upper limbs or femoral approach. Appropriate unfractionated heparin was administered, usually 3000 units for diagnostic procedures and an additional 5000 units for intervention. Contrast medium was injected with an automatic injection device (ACIST CVi®; Dvx Inc., Tokyo, Japan).

### 2.4. Cerebral MRI and Neurological Assessments

An MRI of the brain (1.5-Tesla Achieva Nova Dual, Philips Medical Systems, Netherlands) was performed after the catheterization. The imaging protocol included DW single-shot spin echo-planar [repetition time (TR), 2700 to 4300 ms; echo time (TE), 55 to 84 ms; slice thickness, 5 mm; matrix, 256 × 256; diffusion gradient, *b* values of 0 and 1000 s/mm^2^], fluid-attenuated inversion recovery [FLAIR; TR/TE/inversion time (TI), 8000 to 11000/120 to 125/2400 to 2800 ms], and T2-weighted turbo spin echo (TR/TE, 3700 to 4600/80 to 100 ms) sequences. For each DW sequence, the apparent diffusion coefficient (ADC) map was obtained to exclude false-positive results by a T2 shine-through effect. Acute embolic lesions were defined as focal diffusion abnormalities (bright hyperintense lesions) confirmed on the ADC map. All MRI findings were analyzed by senior radiologists aware of the clinical status and identity of the patients. The localization and number of the lesions were analyzed. We diagnosed acute CI from the combinations of DW imaging and ADC map findings.

### 2.5. Statistical Analysis

Baseline and procedural characteristics of the study population are described as mean ± standard deviation (SD) for quantitative variables and number or percentage for categorical variables. Differences between the CI and non-CI groups were tested by univariate analysis (Pearson's chi-squared test and Welch's *t*-test, as appropriate). Values of *p* < 0.05 were considered statistically significant. All variables with a *p* value <0.05 in the univariate analysis were entered in a nominal logistic regression model by using the forced-entry method because of the small number of patients despite the risk of existing confounding factors. The lack-of-fit test was used to check the adjustment of the model. Nominal logistic regression analysis was used in the multivariate analysis, and odds ratios (ORs) and 95% confidence intervals (95% CIs) were calculated for independent predictors of CI. All statistics were calculated using JMP v10.0.0 (SAS Institute Inc., Chicago, IL, USA).

## 3. Results

### 3.1. Patient and Procedural Characteristics

**Figure 1:**
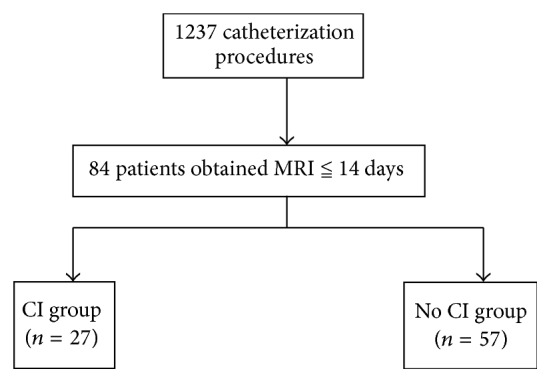
Flowchart of the study population. MRI: magnetic resonance imaging; CI: cerebral infarction.

Among a total of 1237 catheterization procedures, 84 patients (56 men and 28 women; mean age, 71.6 ± 8.7 years) who underwent MRI within 14 days after catheterization (mean duration, 2.81 ± 2.4 days) were included in the study. A flowchart of the study population is shown in Figure 1. DW MRI revealed acute CI in 27 of 84 patients (32.1%). No patient revealed a hemorrhagic lesion. Among 1237 catheterization procedures, 3 patients and 1 patient were clinically diagnosed as stroke and TIA, respectively. We categorized the study subjects into two groups according to the presence or absence of acute CI and compared the patient and procedural characteristics between them (Tables 2 and 3).

Among patient characteristics, patients who exhibited CI on DW MRI were significantly older and had significantly higher rates of dyslipidemia, CAD, and current usage of antiplatelet drugs than patients without CI in univariate analysis. Other factors including hypertension, diabetes mellitus, smoking, renal impairment, decreased LVEF (<40%), severe aortic stenosis, prior myocardial infarction, prior stroke, history of atrial fibrillation, and peripheral vascular disease were not different between the groups (Table 2).

Among procedural characteristics, the number of catheters used (2.85 ± 1.1 versus 2.12 ± 0.9, *p* = 0.004) was significantly greater in the infarction group in univariate analysis. Patients with CI more often underwent procedures in urgent settings (22.2% versus 7%, *p* = 0.05) and for interventional purposes (25.9% versus 8.8%, *p* = 0.04). There were no significant differences in procedural factors, including catheter size, total contrast volume, left ventriculography (LVG), aortography (AoG), and approach sites (Table 3).

In multivariate analysis, age (OR, 1.09; 95% CI, 1.007–1.19; *p* = 0.03), dyslipidemia (OR, 4.46; 95% CI, 1.41–16.03; *p* = 0.01), and number of catheters used (OR, 2.21; 95% CI, 1.21–4.36; *p* = 0.01) were independently associated with acute CI after the procedures (Table 4).

### 3.2. Features of MRI Findings

Features of MRI findings are shown in Table 5. Patients with CI revealed multiple lesions (3.89 ± 4.1). More lesions were observed in bilateral (52%) and anterior plus posterior territories (37%). There was no association among the characteristics of procedures, patients, and lesions.

## 4. Discussion

In this retrospective analysis of catheterization patients in a single institution, we investigated the incidence and risk factors of CI following catheterization. Of note, dyslipidemia, higher age, and number of catheters used were revealed as independent predictors for the incidence of acute CI. Previous studies indicated that impaired cognitive function was associated with cerebral microinfarction as detected with transcranial Doppler ultrasound imaging or DWI [18, 26]. Cerebral microinfarctions correlate with impaired cognitive function and previous studies. To help reduce the occurrence of CI in catheterization patients, these factors need to be considered.

Among baseline and procedural characteristics tested within the present study, dyslipidemia, age, and number of catheters used were identified as independent predictors of CI. Previous studies using DW MRI have reported that possible risk factors are related to atherosclerotic burden and mechanical stress, including hypertension [12], history of vasculopathy [7], number of catheters used [7], fluoroscopy time [7, 9, 10], amount of contrast medium [7], performing internal mammary artery angiography [13], and retrograde passage of the aortic valve [8]. Previous studies using DW MRI found no association between lipid profile and catheterization-related CI [7–14]. Despite the fact that the development of atherosclerotic CAD is associated with LDL, TG, and HDL levels [27], as well as the total level of cholesterol [27, 28], to the best of our knowledge, lipid profile has not been found to have a significant effect on the incidence of CI after catheterization [1, 6, 12]. Unlike other studies that defined dyslipidemia as high total cholesterol (TC) alone [8, 9, 11–15, 29] and as either high TC or statin usage [10], the present study defined dyslipidemia as high LDL level (≥140 mg/dL) or the usage of statin. The result of the multivariate analysis showed dyslipidemia as a predictor of catheterization-related CI (OR, 4.46; 95% CI, 1.41–16.03; *p* = 0.01; Table 4). Meanwhile either high LDL or the usage of statin alone was not significantly different. The possible reason is that patients already using statin were still at higher risk of atherosclerosis. It has been reported that statins were effective for vascular events in patients with aortic plaques [30]. How long the statin needs to be used and what level of LDL should be achieved to reduce catheterization-related CI warrant assessment in future study. Dislodgement of aortic plaque material is assumed to be a source of embolism in patients undergoing catheterization [4], and it has been reported that dyslipidemia is associated with greater aortic plaque thickness in first-ever ischemic stroke patients [31]. Karalis et al. have shown that atherosclerotic aortic debris detected by transesophageal echocardiography is a risk factor for cerebral and peripheral embolism during invasive procedures [32]. Taken together, these results indicate that dyslipidemia associated with increased atheroma burden is a potential risk factor for catheterization-related CI.

Higher age emerged as an independent predictor in the present study. Consistent with our findings, Hoffman et al. have reported that higher age is an independent predictor for PCI-related stroke and TIA [3]. Higher age is a major risk factor for atherosclerotic CAD and development of aortic plaque burden [33–35]. Therefore, higher age is possibly associated with risk of catheter-induced embolization.

Number of catheters used was also an independent predictor for CI in the present study. MRI disclosed that bilateral lesions were present in 52% of the CI group, even though a large proportion of the procedures were performed via either the right or left upper limbs in the present study. Multiple catheter usage might increase the chance of dislodging aortic plaque material, resulting in embolization [7]. It appears likely that the risk of air embolism and dislocation of thrombus from catheters increases with catheter exchanges. Previous studies using DW MRI have identified fluoroscopy time and number of vessels probed as risk factors for CI. These procedural factors could increase the risk of formation of small blood clots and scraping aortic plaques [5, 7, 9, 36]. Continuous heparin flushing might be effective for prevention of intracatheter clot formation. Our findings support that scraping plaques from the ascending aorta and introducing air bubbles with contrast medium are major factors in catheterization-related CI; therefore, careful planning is needed to reduce rates of catheterization-related CI.

LVG did not influence the incidence of CI in the present study. Because it has been assumed that passing through a calcified aortic valve could serve as a source of embolism [8], patients with severely calcified aortic valves or apparent severe aortic stenosis on echocardiography did not undergo LVG in our study.

In the present study, CI was detected in 27 of 84 patients (32.1%) undergoing catheterization. This rate of CI is relatively high compared with previous studies using DW MRI (4.9% to 34.7%) [7–15]. Lund et al. [10] described an incidence of CI of 11.9% in a prospective evaluation with DW MRI, with lower mean age (59.3 ± 9.3 versus 71.6 ± 8.7) and fewer diabetic patients (14.9% versus 42.9%). Meanwhile, Murai et al. [15] reported that acute CI was observed in 34.7% of patients with acute coronary syndrome after primary coronary intervention. The present study also included the patients undergoing urgent catheterization (11.9%). In addition, overall stroke/TIA was observed in 4 of 1237 patients (0.32%) in the present study, the rate of which was almost equivalent to the previous studies [1–3]. Therefore, differences in the demographics of the patients could explain the relatively high incidence of CI in our study.

There are several limitations in the present study. First, the small sample size (*n* = 84), single-center, and retrospective analysis might limit the statistical power and validity of the conclusion. Selection bias also exists in the present study, because MRI was not performed for series of catheterization procedures. Otherwise, since the incidence of catheterization-related CI remains high, 4.9% to 34.7% in other reports [7–15] and 32.1% in our report, this analysis provides insights into reducing the risk of catheterization-related CI. Second, patients did not undergo preprocedural MRI. The patients were determined to have acute CI according to high DWI and low ADC signals. Since it is reported that decreased ADC returns to the baseline after 1–4 weeks [21], subacute CI that developed spontaneously just before the catheterization was not excluded. However, only 3 patients in a total of 580 patients in previous studies displayed acute CI on DW MRI in preprocedural MRI [7–9, 11, 12, 29]. Because of low prevalence of acute CI observed in preprocedural MRI, several studies are performed only after the catheterization like our study [15, 37]. Therefore, a positive finding on a postprocedural MRI strongly indicates acute CI after catheterization.

## 5. Conclusion

Dyslipidemia, age, and number of catheters used appear to be risk factors for catheterization-related CI detected with DW MRI. These findings imply that intervention against dyslipidemia and comprehensive planning to minimize catheter exchanges are important.

## Figures and Tables

**Table 1:** The reasons for MRI in the study subjects.

	*n* = 84
Candidates for coronary artery bypass grafting, %	31 (26)
Candidates for valvular surgery, %	15.8 (13)
Prior syncope, %	11.9 (10)
Assessments for symptoms, %	11.9 (10)
Candidates for pacemaker implantation, %	6 (5)
Candidates for aortic repair, %	6 (5)
Candidates for peripheral vascular surgery, %	3.6 (3)
Others, %	14.3 (12)

**Table 2:** Baseline characteristics of the study subjects.

	All	CI	No CI	*p*
	*n* = 84	*n* = 27	*n* = 57	
Age, yr	71.6 ± 8.7	74.1 ± 6.6	70.4 ± 9.4	0.04^*∗*^
Male, %	61.9	66.7	59.7	0.53
Hypertension, %	73.8	77.8	71.9	0.57
Dyslipidemia (LDL ≧ 140 mg/dL/statin user), %	45.2	63	36.8	0.04^*∗*^
LDL ≧ 140 mg/dL, %	10.7	14.8	8.8	0.46
Diabetes, %	42.9	55.6	36.8	0.11
Smoking, %	59.5	63	57.9	0.66
Family history, %	10.7	11.1	10.5	0.94
Prior stroke, %	14.3	18.5	12.3	0.45
Prior MI, %	26.2	29.6	24.6	0.62
MI within 7 days prior, %	6.0	11.1	3.5	0.17
EF < 40, %	13.1	22.2	8.8	0.09
PVD, %	28.6	40.7	22.8	0.09
CAD, %	60.7	77.8	52.6	0.03^*∗*^
Cre > 1.5 mg/dL, %	14.3	14.8	14.4	0.92
Severe aortic stenosis, %	11.9	3.7	15.8	0.11
AF, %	10.7	14.8	8.8	0.40
Preprocedural medication				
Statin, %	38.1	51.9	31.6	0.09
ACE-I/ARB, %	47.6	51.9	45.6	0.59
Beta blocker, %	29.8	37	26.3	0.32
CCB, %	51.2	55.6	49.1	0.58
Antiplatelet, %	53.6	74	43.9	0.01^*∗*^
Anticoagulation, %	13.1	18.5	10.5	0.31

^*∗*^Included in multivariate analysis.

LDL: low-density lipoprotein; MI: myocardial infarction; PVD: peripheral vascular disease; CAD: coronary artery disease; AF: atrial fibrillation; ACE-I: angiotensin converting enzyme inhibitor; ARB: angiotensin receptor blocker; CCB: calcium channel blocker.

**Table 3:** Procedural characteristics.

	All	CI	No CI	*p*
	*n* = 84	*n* = 27	*n* = 57	
No. of catheters used	2.35 ± 1.0	2.85 ± 1.1	2.12 ± 0.87	0.004^*∗*^
Catheter size, Fr	4.31 ± 0.66	4.44 ± 0.80	4.25 ± 0.58	0.25
Contrast volume, mL	122.8 ± 55	128 ± 55	120 ± 55	0.57
Fluoroscopy time, min	18.9 ± 13.1	22.7 ± 14	17.1 ± 12.3	0.08
LV angiogram, %	45.2	48.2	43.9	0.71
Ao angiogram, %	13.1	11.1	14.0	0.71
Urgent, %	11.9	22.2	7.0	0.05^*∗*^
IABP, %	1.2	3.7	0	0.14
Purpose of procedure				
Diagnostic, %	85.7	74.1	91.2	0.04^*∗*^
Interventional, %	14.3	25.9	8.8	0.04^*∗*^
Approach site				
Upper limbs, %	81.0	81.5	80.7	0.93
Femoral, %	19.0	18.5	19.3	0.93

^*∗*^Included in multivariate analysis.

LVG: left ventriculography; AoG: aortography; IABP: intra-aortic balloon pumping.

**Table 4:** Multivariate analysis to determine factors associated with acute CI after catheterization.

	OR	95% CI	*p*
Age (increase per 1 year)	1.09	1.007–1.19	0.03^*∗*^
Dyslipidemia (high LDL/statin user)	4.46	1.41–16.03	0.01^*∗*^
CAD	1.64	0.42–6.67	0.47
Antiplatelet use	1.69	0.46–6.33	0.42
Number of catheters used (increase per 1 catheter)	2.21	1.21–4.36	0.01^*∗*^
Urgent	3.43	0.61–22.84	0.16
Interventional	1.27	0.24–6.4	0.77

Lack-of-fit test			0.43 (Prob > *χ* ^2^)

^*∗*^Independent predictor for CI.

CAD: coronary artery disease.

**Table 5:** Features of MRI findings.

	Patients with CI
	*n* = 27 (%)
Lesions per patient	3.89 ± 4.1
Patients with a single lesion	9 (33)
Patients with multiple lesions	18 (67)

Lesion location, patients	
Right hemisphere	6 (22)
Left	7 (26)
Bilateral lesions	14 (52)
Anterior circulation territory	10 (37)
Posterior	7 (26)
Anterior and posterior	10 (37)
